# The Tenebrionidae of California: A Time Sensitive Snapshot Assessment

**DOI:** 10.3897/zookeys.415.6523

**Published:** 2014-06-12

**Authors:** Rolf L. Aalbu, Aaron D. Smith

**Affiliations:** 1 Department of Entomology, California Academy of Sciences, 55 Music Concourse Dr., Golden Gate Park, San Francisco, California, 94118; 2 Department of Biological Sciences, Northern Arizona University, PO Box 5640, Flagstaff, AZ, 86011-5640, USA

**Keywords:** California, Floristic Regions, Tenebrionidae, Biodiversity, Hotspots, Conservation

## Abstract

Due to a diversity of habitats and its geologic history, the US state of California hosts a spectacular assemblage of darkling beetle species (Coleoptera: Tenebrionidae). In addition to being part of the California Floristic Province, one of 34 global biodiversity hotspots identified by Conservation International, California also has additional areas which are parts of the Great Basin, Mojave, and Sonoran deserts. California is divided into nine floristic regions. Each region is assessed in terms of faunal composition and endemism. A “snapshot” of our present knowledge of the Tenebrionidae indicates that 447 currently recognized species, representing 108 genera, occur in California of which one hundred and ninety are endemic. California is compared to other nearby regions in diversity and endemism. An analysis of currently valid species vs a more realistic species account based on unpublished records of likely synonyms and known species yet to be described in the scientific literature is presented. The California Floristic Region, rather than other more arid parts of California, has the highest number of total and endemic species. Because of their high diversity and endemism, tenebrionids could potentially provide a valuable tool for monitoring the environment for conservation purposes.

## Introduction

The state of California is part of the California Floristic Province, one of 34 global biodiversity hotspots identified by Conservation International^1^. Over 50 percent of the world’s plant species and 42 percent of all terrestrial vertebrate species are endemic to these 34 biodiversity hotspots, a total area which covers only 2.3 percent of the Earth’s land surface. The California Floristic Province includes most of western California and a small section of Baja California and Southwestern Oregon. On Conservation International’s California Floristic Province website, although numbers of endemic plants, birds, mammals and amphibians are listed, nothing is mentioned concerning insects^2^. California also includes areas not considered to be part of the California floristic province. These areas contain aspects of the Great Basin, Mojave, and Sonoran deserts.

In 2010 the Essig Museum of Entomology at University of California, Berkeley began CalBug (NSF-DBI: 0956389), a collaborative project among nine California museums with a goal to digitize and geographically reference over one million specimens from target groups and localities^3^. Tenebrionidae was one of the focus groups in Coleoptera. However, to date, few tenebrionids (2%) have been digitized and georeferenced, all at Santa Barbara Museum of Natural History (SBMNH).

**Figure 1.**
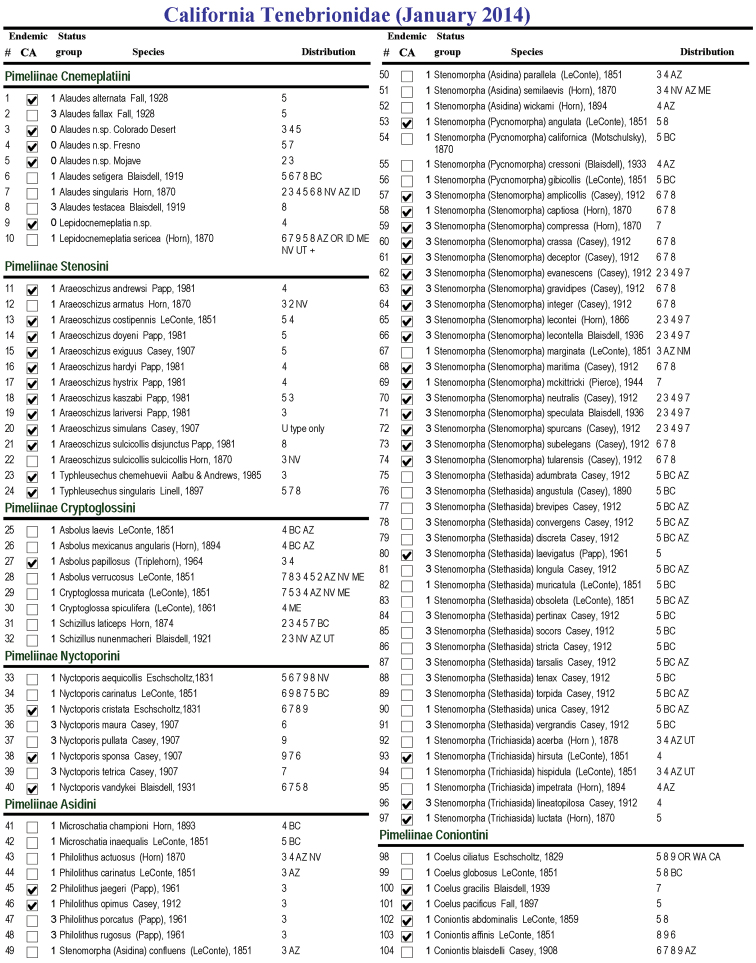

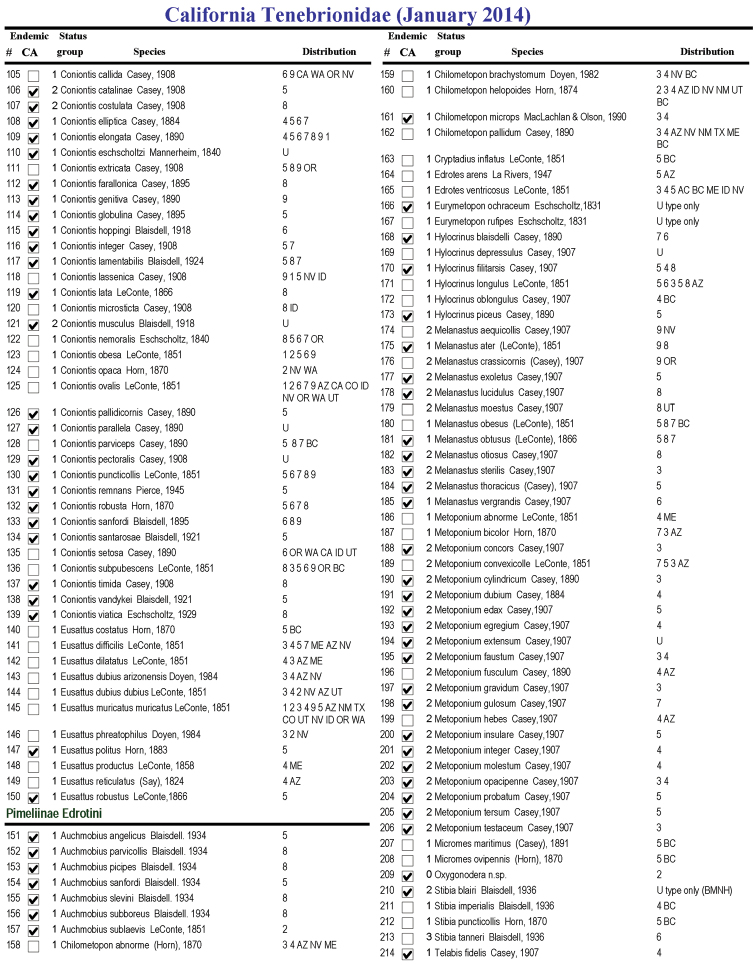

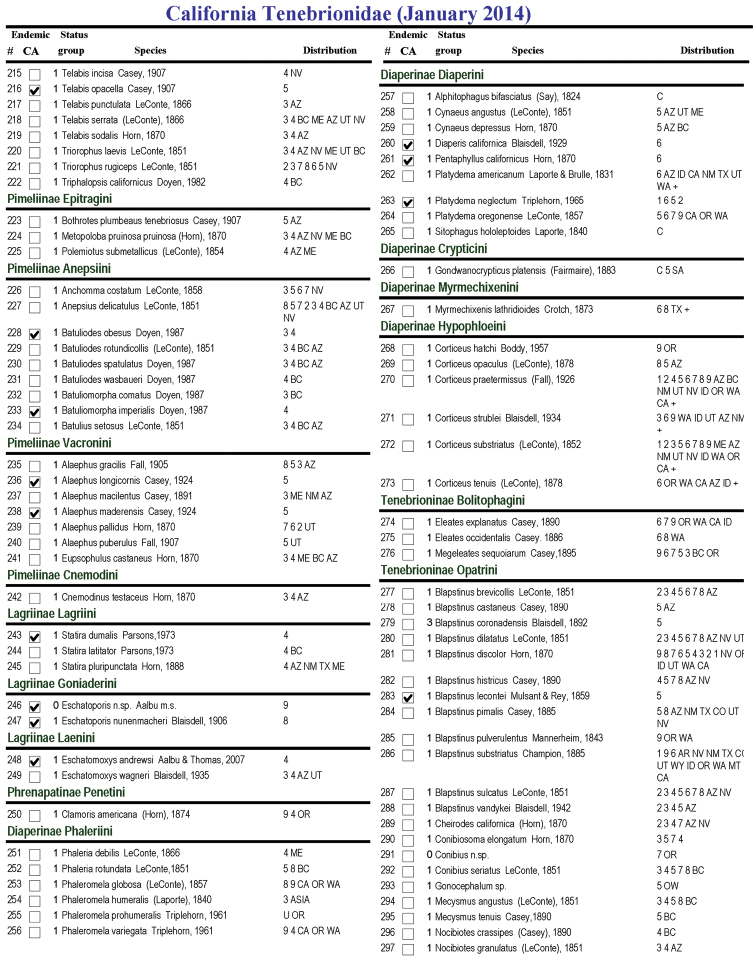

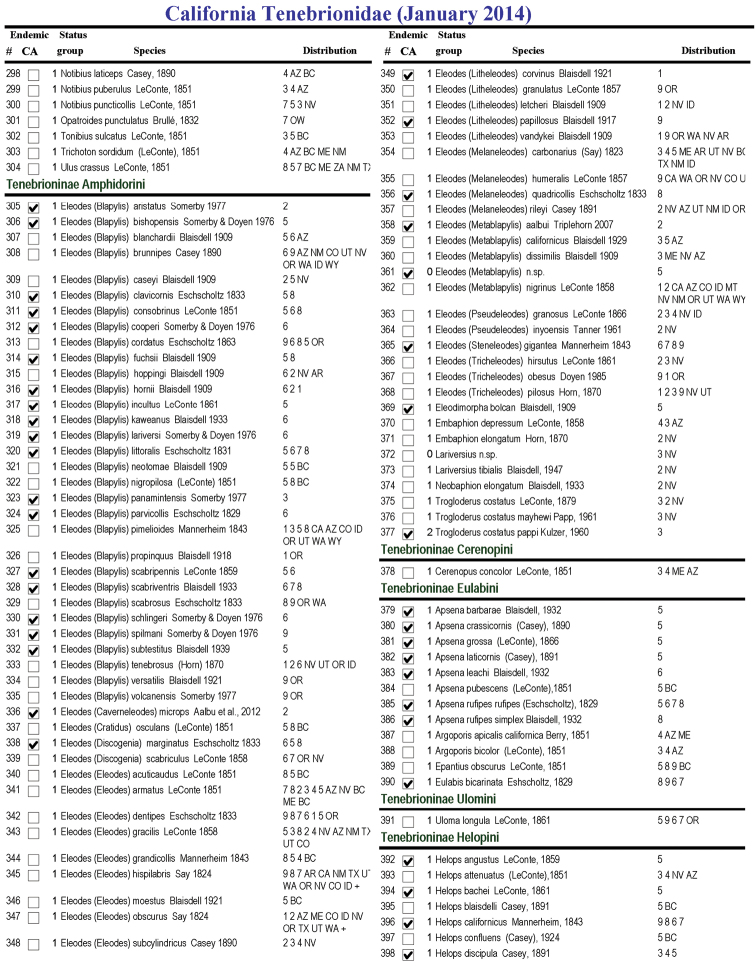

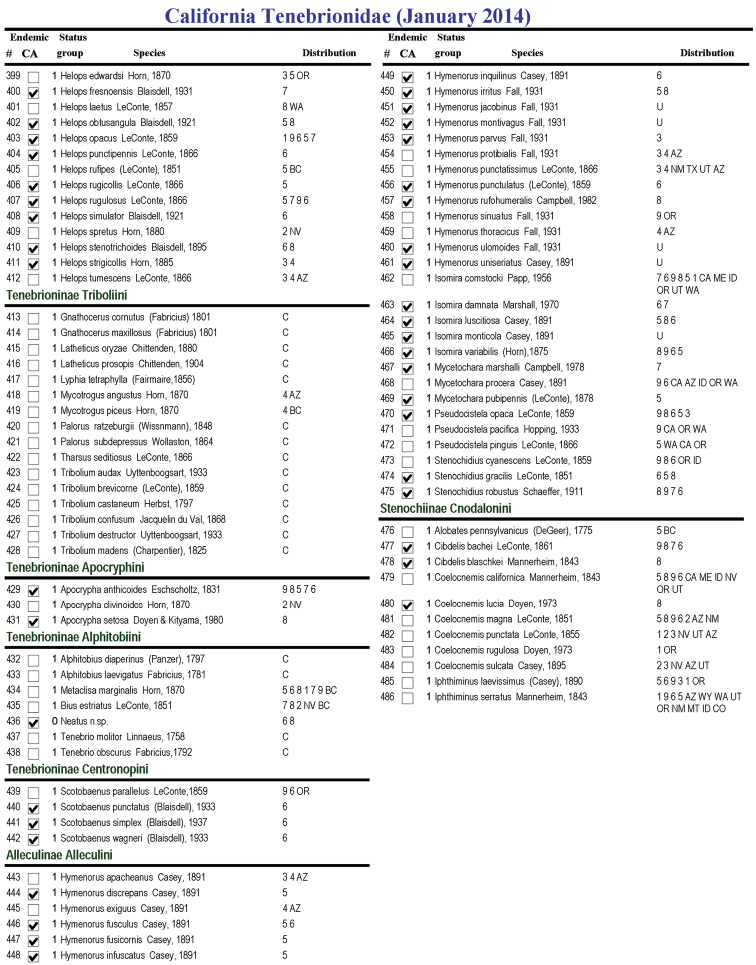
Checklist of the California Tenebrionidae species with distributions and likelihood for future synonymy. Distribution numbers refer to California regions (Fig. 2) and the following: **ME** (Mexico) **BC** (Baja California) **NV** (Nevada) **AZ** (Arizona) **ID** (Idaho) **UT** (Utah) **NM** (New Mexico) **OR** (Oregon) **WA** (Washington) **CA** (Canada) **U** (unknown California distribution) **C** (refers to cosmopolitan pest), ASIA **SA** (South America), and **OW** (Old World).

In 2005 Mike Caterino, formerly at SBMNH, solicited the author’s help in contributing to a web accessible list of “Beetles of California”. This was followed by a visit to the SBMNH in 2007 to provide additional identifications of beetles in the collection. The list, last updated in 2009, is posted on http://www.sbnature.org/collections/invert/entom/cbphomepage.php [accessed on December 9, 2013]. An updated list is present here (Fig. 1) that reflects a current “snapshot” of our knowledge of this fauna. It is also available online (http://insectbiodiversitylab.org/CaliforniaDarklingBeetles.html). To account for active research and our growing understanding of the California fauna, the list includes a separate column assessing the potential that each species will be synonymized in future works (see below). Both the current valid species list and a list excluding likely synonymous species, but including known undescribed species, are analyzed based on each species’ known occurrence in each of California’s nine floristic provinces to assess number of tenebrionid species in each province and their endemicity.

## Materials and methods

Sources of Information other than the SBMNH list above include publications from early workers (LeConte, Horn, Motschulsky, Casey, Blaisdell, and others), modern workers (Doyen, Triplehorn, Somerby, Brown, Smith, and others)^4^, and modern revisions: Parts of the Coniontini (Doyen 1984), Cnodalonini (Doyen 1973), Amphidorini (Aalbu et al. 2012, Triplehorn and Thomas 2011), Edrotini (Pape et al. 2007), Stenosini (Papp 1981) and Asidini (Brown and Doyen 1991, Smith 2013) as well as complete revisions of the Cryptoglossini (Aalbu 2005) and Anepsiini (Doyen 1987). Other major sources of information include the Species Database of the California Academy of Sciences and information from the author’s personal collection (the Rolf L. Aalbu Collection – RLAC), as well as visits to all major beetle collections in California and many others outside of the state. Information for potential future species synonymies and undescribed species come from the authors’ research, discussions with other tenebrionid workers, and currently unpublished studies by the authors, Ron Somerby, and Charles Triplehorn.

To account for the many groups in which data has been accumulated but no recent revision has been published, the Tenebrionidae records from California were categorized in the following status groups based on their current and future status: 0), Known new but undescribed species; 1), Currently projected valid species and subspecies^5^; 2) Most likely synonyms, but synonymy not determined without further study; and 3), Known but unpublished synonyms. Published synonyms were omitted. The assessment was then divided into two categories: A.) Described Species Count: All species currently valid in the literature including known synonyms (groups 1, 2, and 3 above). B), Realistic Species Count: (groups 0, 1, and 2 above). Endemism was calculated on a strict basis (species endemic to specific regions which include parts of adjacent areas not in California were not considered).

For the purpose of this study, California is divided into nine floristic regions modified from a map by the Jepson Herbarium^6^ (Fig. 1). Four of these are not considered parts of the California Floristic Region. These are: Region 1, The Northern Great Basin Province, including the Warner Mountains and Modoc Plateau; Region 2, The Southern Great Basin Province, including the White and Inyo Mountains and intermountain valleys east of the Sierras Nevada’s and White Mountains; Region 3, The Mojave Desert and associated desert mountains; and Region 4, The Sonoran (Colorado) Desert and associated desert mountains.

Regions belonging to the California Floristic Region include: Region 5. The South Coast, including the Transverse and Peninsular Ranges and Channel Islands; Region 6, The Sierra Nevada Mountains; Region 7, The Central Valley; Region 8, The Central Coast, including the San Francisco Bay area and Coast Ranges; and Region 9, The Northern Coast, including the Cascade and Klamath Ranges as well as the Northern Coast Ranges. In these regions we examined species occurrence and regional endemism. Regional endemism was also calculated on a strict basis as described above.

## Results and discussion

It is important to keep in mind that this study represents a snapshot in time and thus is subject to change as new information becomes available. However, this assessment is also a balance between future synonymies from previous descriptions (Casey and other early workers: *Coniontis*, various genera of edrotines) on one side and new species discoveries, as well as new foreign introductions, on the other. At present, we know of at least eight distinct new species.

A list of all described species is presented in phylogenetic order (Fig. 1). Differences in group numbers and endemics are presented in Table 1. Differences in species count categories (numbers, endemics and percent endemism) are shown in Table 2. It is notable that despite the differences in numbers, both analyses (described vs realistic) indicate a very similar percent endemism. Since this study is intended as a “snapshot” of our current knowledge, species counts and analysis, unless otherwise specified, include only groups 0, 1, and 2 (Realistic Species Count). This tenebrionid inventory of California thus includes 34 tribes, 118 genera and subgenera, 447 species and subspecies (including known new species). Of these, 190 are endemic to California. The present SBMNH web list includes 471 species from California. Of these, 10 are collection data errors. These included *Argoporis alutacea* Casey; *Asidopsis consentanea* Casey; *Asidopsis planata* (Horn); *Cryptoglossa variolosa* Horn; *Eleodes alticola* Blaisdell; *Eleodes subnitens* LeConte; *Neatus tenebrioides* Beauvois; *Platydema micans* Zimmerman; and *Stenomorpha obovatus* (LeConte) none of which are known to occur in California. Others are known but unpublished synonymies (status group 3).

**Table 1.** Status Groups and Endemicity. Group 0: Known new but undescribed species; Group 1: currently projected valid species and subspecies; Group 2: most likely synonyms, but synonymy not determined without further study; and Group 3: known but unpublished synonyms.

Status group	Non endemic species	Endemic species	Total
0	2	8	10
1	249	155	404
2	6	27	33
3	22	17	39
	279	207	486

**Table 2.** California Species, Described vs Realistic. Species counts for the state and % endemicity based on current valid species (A) and a realistic estimation of actual species counts (B).

Category	Status groups	Species	Endemics	Total	% Endemic
A: Described	1, 2 & 3	277	199	476	41.81%
B: Realistic	0, 1, & 2	257	190	447	42.51%

The fauna is composed of the following subfamilies in descending species number: Pimeliinae (204), Tenebrioninae (168), Alleculinae (33), Diaperinae (23), Stenochiini (11), Lagriinae (7), and Phrenapatinae (1). California is clearly a center of diversity for the family Tenebrionidae, representing 38% of all U.S. species. The most abundant tribes and genera in terms of species numbers are: Amphidorini (73 species), Edrotini (71 species), Coniontini (53 species), Alleculini (33 species), Opatrini (26 species), Asidini (25 species), and Helopini (21 species); and genera such as *Eleodes* (64 species), *Stenomorpha* (19 species), *Coniontis* (38 species), and *Metoponium* and *Helops* each with 21 species. A number of tribes such as Amphidorini, Coniontini, and Nyctoporini, and genera such as *Eleodes*, *Coelocnemis*, *Nyctoporis*, *Asbolus*, *Coniontis*, and *Alaudes* also exhibit their greatest diversity in genera/species in California.

Compared to other known nearby geographical regions, California also has a high species per area diversity (1.05 per 1000 square miles) which is higher than the U.S. as a whole^7^ (.12) or even Mexico^7^ (.68), but not Baja California^7^ which has a species diversity of 5.47 (see Table 3). California shares species with the following adjoining areas in descending order: 1. Southwest U.S.: (including Arizona, 101, Nevada, 76; New Mexico, 23; and Utah, 42). 2. Mexico (mainland 32, Baja California, 68) and 3. Northwest U.S. (including Oregon, 56; Washington, 33; and Idaho, 32. A number of species are known only from the type and have undetermined California localities (16). Twenty species are cosmopolitan pests. See Fig. 1 for additional locality information.

**Table 3.** Comparison of currently valid species/endemics per area for various regions.

Region	Number of species	Number of endemics	% Endemism	Area (km^2^)	Species diversity per 1000 km^2^
California*	447**	190	43%	423970	1.05
USA***	1184	?	>60%	9827000	0.12
Mexico***	1340	723	54%	1973000	0.68
Baja California***	404	225	56%	73909	5.47

* Bordered by 3 states and Baja California.

** 34% of all U.S. species.

*** numbers probably 5–8 years old.

The distribution of California tenebrionids can be divided into six patterns: 1), Widespread species, 2), Restricted but not especially hard to collect species (Caves, single canyons (*Eschatomoxys andrewsi* Aalbu & Thomas, *Eleodes (Caverneleodes) microps* Aalbu et al.), 3), Restricted but very difficult to collect species (*Eleodimorpha*, *Oxygonodera*), 4), Historically abundant but now difficult to collect species (*Eleodes (Melaneleodes) quadricollis* Eschscholtz), 5) Introduced species composed of standard stored product pests as well as other introductions not associated with stored products (*Opatroides punctulatus* Brullé and *Gonocephalum* sp.) and 6) species only known form the type material with specific locality unknown. California also has some unusual darkling beetle occurrences and absences compared to the rest of North America. One is the presence of two species from the Asian tribe Laenini, which is otherwise absent on the continent. Another is the absence of the genus *Strongylium*, a species-rich genus found worldwide including in Arizona (2 species) and most of the rest of the United States.

## Regional analysis

**Figure 2.**
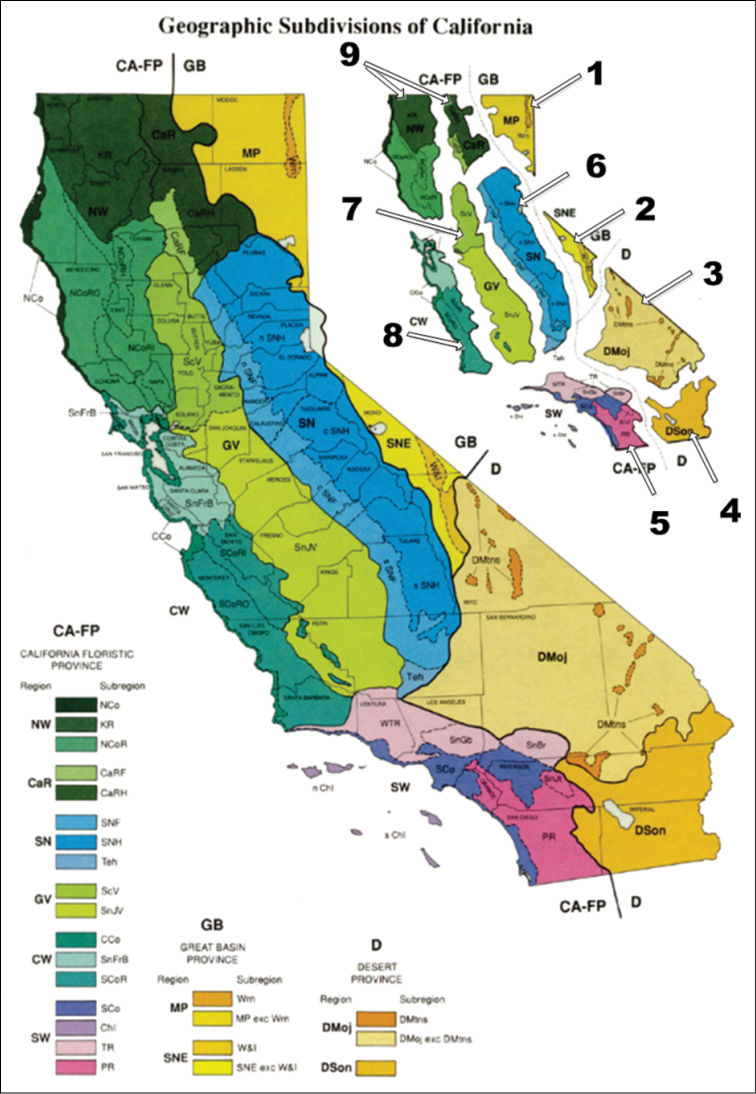
Geographic subdivisions of California from http://ucjeps.berkeley.edu/cguide.html#Map with Unit Boundaries with regions 1–9 outlined.

**Figure 3.**
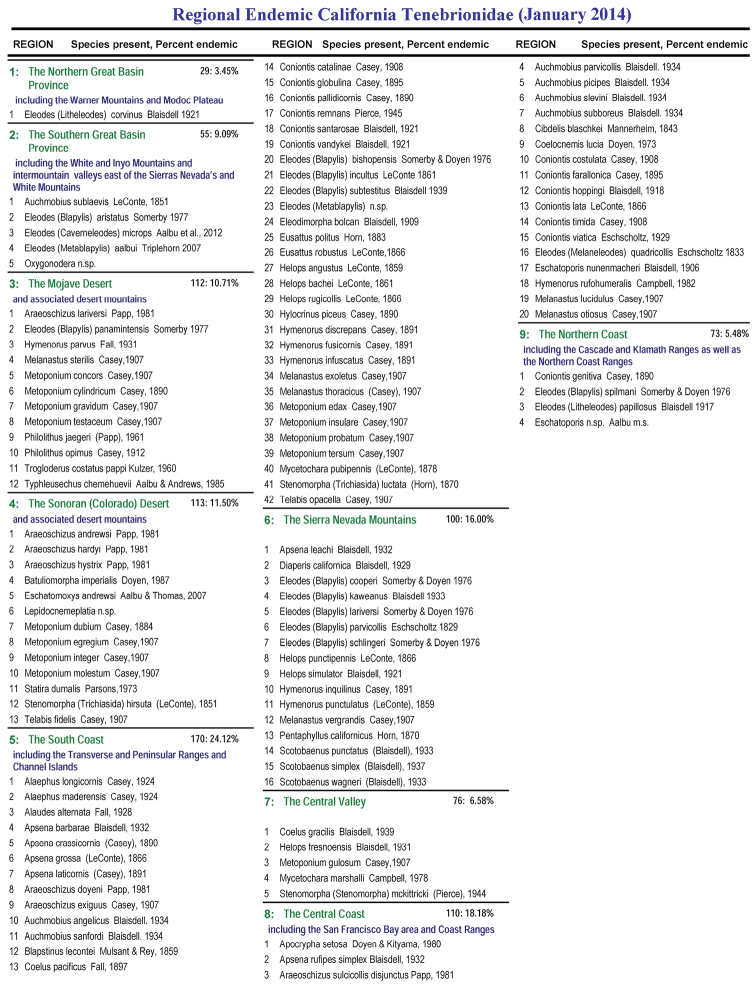
Regional Endemic California Tenebrionidae.

For the purpose of this study, California was into 9 floristic regions (Fig. 2) to examine species occurrence and regional endemism. Regional endemism was also calculated on a strict basis as mentioned above. A list of all regional endemics is presented as well as total species numbers for the region and percent endemism (Fig. 3). These areas are ranked in Table 4. Adding the above data suggests that over 62% (62.11) of the endemic species in California are regional endemics while 43% (42.60) of all tenebrionids are endemic in terms of being regional endemics or multiple region endemics.

**Table 4.** Comparison of regional endemics and all endemics for California.

Region	Endemic species	All species	% Endemic	% of all California Endemics
5. South Coast & Islands	42	171	24.56%	35.59%
8. Central Coast & Bay	20	110	18.18%	16.95%
6. Sierra Nevada	16	100	16.00%	13.56%
4. Sonoran Desert	13	113	11.50%	11.02%
3. Mojave Desert	12	112	10.71%	10.17%
8. Central Valley	5	76	6.58%	4.24%
2. South Great Basin	5	55	9.09%	4.24%
9. North Coast	4	73	5.48%	3.39%
1: North Great Basin	1	29	3.45%	0.85%

One may note that, somewhat surprisingly, subregions within the California Floristic Region have more regional endemic species (87) as well as California endemic species (124) despite the common association of tenebrionids with desert habitats, where they are always abundant (see Table 5). On this table, “all endemics” in the “unknown….” region refer to species where the type locality is simply listed as “California”. This “snapshot” assessment emphasizes how much remains to be done in this area, especially in revising tribes or genera which have not been looked at since their description, as well as rediscovering species of “unknown” California localities. Additional new species, as well as new introductions, will undoubtedly be discovered as well. It is hoped that this type of assessment can be useful in environment monitoring and conservation studies.

**Table 5.** Comparison of species endemicity for California Floristic affinities.

Floristic Region	All Endemics	Non Endemic	All Species
Desert Areas	37	94	131
California Floristic Province	124	81	205
Both Areas	16	60	76
Unknown California locality, cosmopolitan or introduction	13	22	35

## Footnotes

^1^ See http://www.conservation.org/where/priority_areas/hotspots/Pages/hotspots_main.aspx [accessed on December 9, 2013].

^2^ See http://www.conservation.org/WHERE/PRIORITY_AREAS/HOTSPOTS/NORTH_CENTRAL_AMERICA/CALIFORNIA-FLORISTIC-PROVINCE/Pages/default.aspx [accessed on December 9, 2013].

^3^ See http://calbug.berkeley.edu/data.html [accessed on December 9, 2013].

^4^ All publications prior to 2002 are listed in Aalbu et al. 2002. Newer pertinent publications are listed in the reference section below.

^5^ Some of the early described species as Edrotines etc., described by early workers as Casey may potentially be synonyms.

^6^ Geographic subdivisions of California, Jepson Flora Project (eds.) [2013] Jepson eFlora, http://ucjeps.berkeley.edu/IJM.html [accessed on December 9, 2013].

^7^ Numbers probably 5–8 years old.
